# Caloric restriction and time-restricted feeding differentially modulate metabolic, adipose tissue, and neurobehavioral outcomes in obese rats fed a high-sugar diet

**DOI:** 10.3389/fnut.2026.1933559

**Published:** 2026-10-29

**Authors:** Hassler Clementino Cavalcante, Danielle Melo de Souza, Maria Letícia da Veiga Dutra, Mariany Bernardino Barbalho Fonte, Aline Barbosa D'oliveira, Kamila Sabino Batista, Guilherme Costa de Oliveira, Rafaela Lira Formiga Cavalcanti de Lima, Lucas Rannier Ribeiro Antonino Carvalho, Alana Natalícia Vasconcelos de Araújo, Naís Lira Soares, Estefanía Farías-Gaytán, Omar Guzman-Quevedo, Adriano Francisco Alves, Jailane de Souza Aquino

**Affiliations:** 1 Department of Nutrition, Laboratory of Experimental Nutrition, Campus I, Federal University of Paraíba (UFPB), João Pessoa, Brazil; 2 Postgraduate Program in Nutritional Sciences, Campus I, Federal University of Paraíba (UFPB), João Pessoa, Brazil; 3 Secretariat for Health and Environmental Surveillance, Ministry of Health, Brasília, Brazil; 4 Department of Nutrition, Laboratory of Bromatology, Campus I, Federal University of Paraíba (UFPB), João Pessoa, Brazil; 5 Department of Physiology and Pharmacology, Karolinska Institutet, Biomedicum, Solna, Stockholm, Sweden; 6 Programa Institucional de Doctorado en Ciencias Biológicas (Opción en Biotecnología Molecular), Universidad Michoacana de San Nicolás de Hidalgo, Morelia, Michoacán, Mexico; 7 Laboratory of Experimental Neuronutrition and Food Engineering, Tecnológico Nacional de México (TECNM)/Instituto Tecnológico Superior de Tacámbaro, Tacámbaro, Michoacán, Mexico; 8 Pathology Laboratory, Department of Physiology and Pathology, Campus I, Federal University of Paraíba (UFPB), João Pessoa, Brazil

**Keywords:** anxiety-like behavior, caloric restriction, depression-like behavior, insulin resistance, interleukin 6, obesity, time-restricted feeding

## Abstract

**Introduction:**

This study aimed to compare the effects of time-restricted feeding and caloric restriction on glycemic control, adipose tissue IL-6 immunoreactivity, and behavioral outcomes in rats with obesity induced by a high-sugar diet.

**Methods:**

A total of 40 adult male Wistar rats (approximately 80 days old) were randomized into two groups (initially): a control group (CG, *n* = 10), fed a standard diet, and an obese group (OG, *n* = 30), fed a high-sugar diet (HSD) for 13 weeks to induce obesity. Subsequently, animals were randomized into four experimental groups: control (CG, *n* = 10), obese (OG, *n* = 10), obese with caloric restriction (OCR, *n* = 10), and obese with time-restricted feeding (OTRF, *n* = 10), and subjected to their respective interventions for 4 weeks.

**Results:**

OCR produced the most pronounced metabolic benefits, significantly reducing body weight, the area under the curve (AUC) of the insulin tolerance test (ITT), insulin resistance, fasting glucose, insulin and leptin levels, adiposity index, adipocyte area, and IL-6 immunopositive area (all *p* < 0.05), while increasing locomotor and exploratory behavior. Conversely, OTRF primarily modified behavioral outcomes, with increased swimming and reduced immobility in the forced swim test, and also reduced insulin resistance, fasting glucose, insulin and leptin levels (all *p* < 0.05). OTRF also reduced adipose tissue IL-6 immunoreactivity but did not significantly reduce adipocyte size. Localized hippocampal morphological alterations were observed in OTRF rats.

**Discussion:**

Caloric restriction was more effective in improving metabolic outcomes, whereas TRF primarily modified behavioral parameters, indicating distinct responses to these dietary interventions.

## Graphical Abstract

**Figure:**
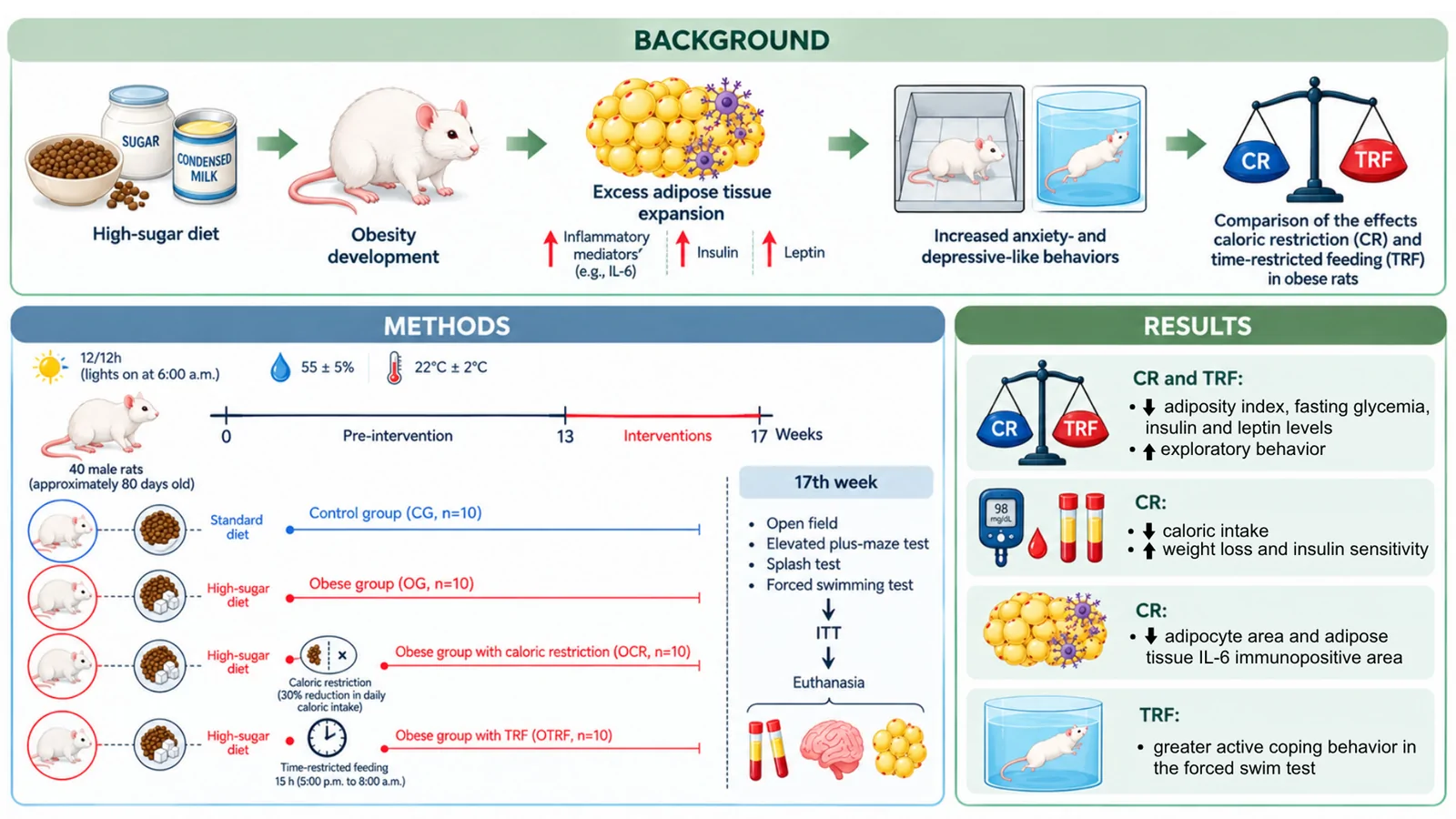

## 1 Introduction

Obesity is a chronic disease characterized by excessive accumulation of body fat resulting from an imbalance between caloric intake and energy expenditure, arising from the interaction of genetic, hormonal, microbial, and environmental factors, among others (1). Its occurrence is strongly associated with the development of other noncommunicable chronic diseases (NCDs), including diabetes, cardiovascular diseases, and cancer (2).

Despite global efforts to curb the rise in obesity, its prevalence has increased substantially, reaching pandemic proportions and imposing a major burden on individuals, society, and healthcare systems (3). It is estimated that by 2050, approximately 1.95 billion individuals over 25 years of age will be living with obesity worldwide, representing an escalating challenge for public health systems (4).

The increased consumption of ultra-processed foods over recent decades, driven by changes in the global food system, has been consistently associated with the growing incidence and prevalence of excess body weight (5). Indeed, previous studies in both animals (6–8) and humans (9, 10) have demonstrated that the consumption of diets rich in fat and sugar contributes to the development of metabolic disorders such as obesity and diabetes (11–13).

In obesity, excessive expansion of subcutaneous and visceral adipose tissue, driven by adipocyte hypertrophy (increased cell size) and hyperplasia (recruitment of new adipocytes), triggers a chronic low-grade inflammatory state (14). This condition is characterized by infiltration of pro-inflammatory immune cells and local production of inflammatory cytokines, including interleukin 1β (IL-1β), interleukin 6 (IL-6), and tumor necrosis factor-alpha (TNF-α), which contribute to metabolic disturbances such as hyperinsulinemia and hyperleptinemia, thereby exacerbating the clinical manifestations of obesity (15, 16).

Among the inflammatory mediators associated with obesity, interleukin-6 (IL-6) has emerged as an important regulator of adipose tissue dysfunction and metabolic impairment. Increased adipose tissue IL-6 expression has been associated with obesity-related insulin resistance, impaired glucose homeostasis, and chronic low-grade inflammation (17). Given its close association with metabolic dysfunction, adipose tissue IL-6 has been proposed as a relevant biomarker for evaluating inflammatory responses to dietary interventions in obesity (18, 19).

Insulin resistance represents one of the earliest and most clinically relevant metabolic disturbances associated with obesity and is considered a major link between adipose tissue dysfunction and the development of type 2 diabetes. Adipose tissue-derived inflammatory mediators, including IL-6, contribute to impaired insulin signaling, disrupted glucose homeostasis, and alterations in endocrine regulators such as insulin and leptin, thereby promoting metabolic dysfunction (18, 19).

Furthermore, this inflammatory milieu has been associated with microglial activation and exacerbated neuroinflammation, processes linked to synaptic remodeling, neurodegeneration, and cognitive dysfunction. These alterations particularly affect brain regions such as the hippocampus, contributing to increased anxiety- and depression-like behaviors (20–22).

In this context, non-pharmacological interventions such as caloric restriction (CR) have gained considerable attention in obesity management because of their effectiveness in reducing body weight and promoting metabolic health and are widely considered the gold standard for weight-loss interventions (23). However, long-term adherence to CR remains limited due to difficulties in maintaining dietary restriction and the high rates of treatment discontinuation (24).

As an alternative dietary strategy, intermittent fasting (IF) has been increasingly investigated because of its promising effects on body weight control and glycemic regulation in individuals with obesity (25). Among the different IF protocols, time-restricted feeding (TRF; referred to as time-restricted eating, TRE, in human studies) has emerged as a particularly attractive approach because it allows *ad libitum* food intake within feeding windows ranging from 4 to 12 h, without explicit restrictions on total caloric intake or diet quality, unlike CR (24, 26). In rodent models, TRF has been shown to reduce caloric intake, inflammatory markers, body weight, and fat accumulation, while also preventing behavioral alterations and improving glucose tolerance and insulin sensitivity in male animals (27–29). However, it remains insufficiently understood whether the effects of TRF are comparable to those of CR in the management of obesity induced by high-sugar diets (8).

We hypothesized that TRF would improve metabolic homeostasis and neurobehavioral outcomes while attenuating systemic and central inflammatory responses, producing effects comparable to or greater than those achieved with CR in rats with diet-induced obesity. Therefore, the present study aimed to compare the effects of TRF and CR on metabolic, inflammatory, and neurobehavioral outcomes in rats with obesity induced by a high-sugar diet.

## 2 Materials and methods

### 2.1 Animals, diets and experimental protocol

Forty male Wistar rats (*Rattus norvegicus albinus*), approximately 80 days old and with an initial mean body weight of 353.8 ± 7.9 g, were obtained from the José Paulino Animal Facility of the Department of Nutrition at the Federal University of Pernambuco (UFPE) and acclimatized for 1 week prior to the biological assay. The rats were housed in polypropylene cages (five animals per cage) equipped with grid flooring, as previously described by Soares et al. (2021) (30). Rats were maintained under standard environmental conditions, including a 12 h light/dark cycle (lights on at 6:00 a.m.), controlled temperature (22 ± 2 °C), and relative humidity (55 ± 5%), with free access to water.

All experiments were conducted at the Experimental Nutrition Laboratory (LANEX-UFPB, Paraíba, Brazil) following approval by the Animal Ethics Committee of the Federal University of Paraíba (protocol no. 5752150319/2019), in accordance with the ARRIVE Guidelines for Reporting *In Vivo* Animal Experiments (31).

Following the acclimatization period, body weight was recorded for all rats and ranked from lowest to highest. A weight-balanced randomization procedure based on initial body weight was then used to allocate the animals to either a control group (CG, *n* = 10), fed a standard commercial diet (Purina^®^, Paulínia, Brazil), or an obese group (OG, *n* = 30), fed a high-sugar diet (HSD) for 13 weeks to induce obesity. This procedure was used to minimize baseline differences in body weight between groups. After the 13-week obesity-induction period, the body weights of the obese animals were reassessed, and the same weight-balanced randomization approach was used to allocate them to three experimental groups: obese (OG, *n* = 10), obese with caloric restriction (OCR, *n* = 10), and obese with time-restricted feeding (OTRF, *n* = 10). All obese groups continued to receive the HSD throughout the intervention period (weeks 14–17), whereas the control group remained on the standard diet throughout the study.

The time-restricted feeding (TRF) protocol was selected based on previous rodent studies (8, 32, 33), considering its broad clinical application for the prevention of metabolic disorders without altering total caloric intake (34, 35). For implementation, rats in the OTRF group underwent a daily fasting window of 15 h (from 5:00 p.m. to 8:00 a.m.) with *ad libitum* access to food during the remaining 9-h period (from 8:00 a.m. to 5:00 p.m.) over the final 4 weeks (weeks 14–17) of the experimental protocol. In contrast, animals in the OCR group were subjected to a 30% caloric restriction based on the mean food intake of the control group (8) (Figure 1).

**Figure 1:**
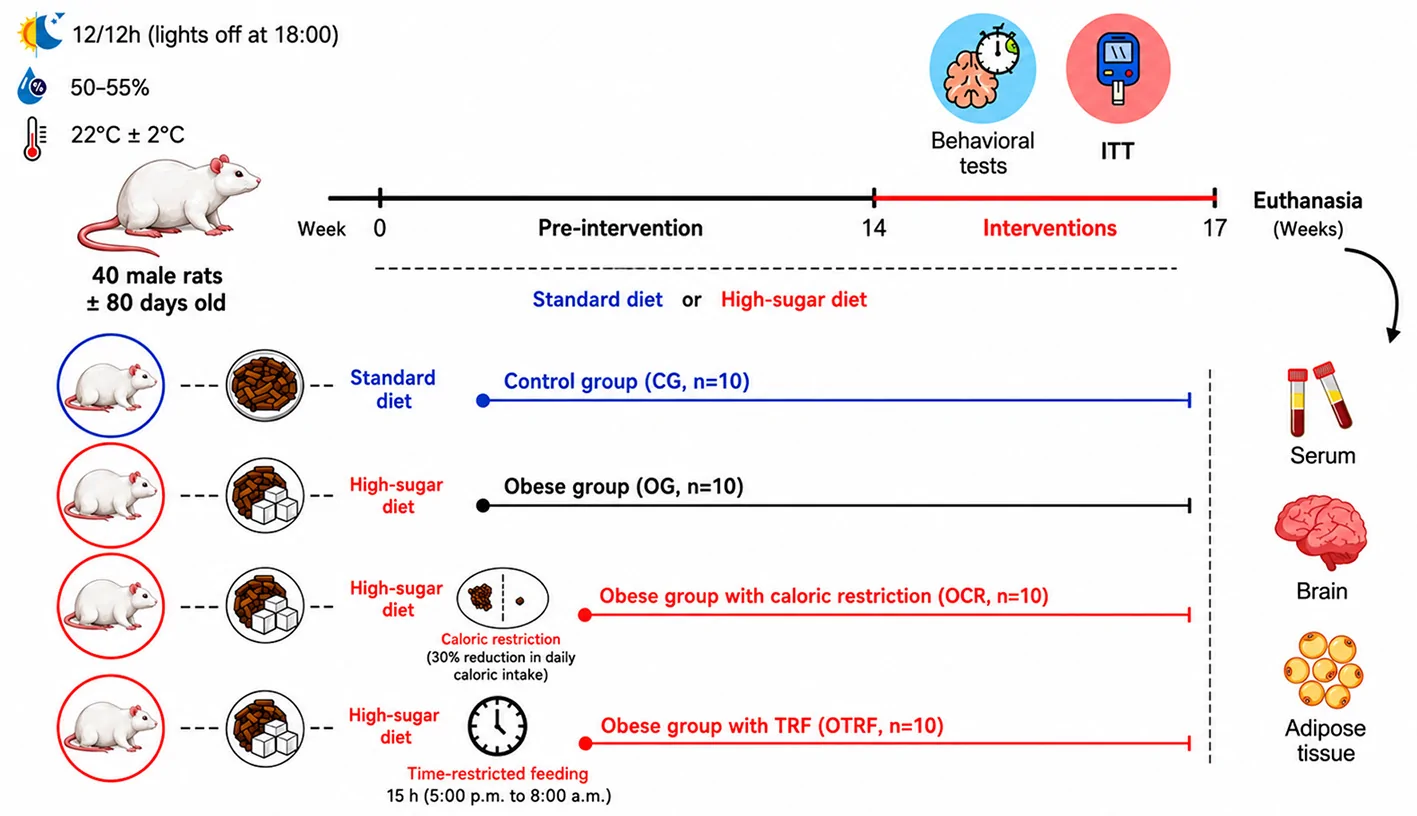
Experimental design of the study. ITT, insulin tolerance test.

Diets were previously analyzed for proximate composition (36) and sugar profiles by high-performance liquid chromatography (1260 Infinity LC system, Agilent Technologies, Santa Clara, CA, USA) (8) (Supplementary Table 1). Body weight and food intake were recorded weekly, and energy intake was calculated based on food consumption (g) and diet composition.

### 2.2 Behavioral tests

All behavioral assessments were conducted at the beginning of the dark phase (6 p.m.) in a quiet experimental room maintained at 22 ± 2 °C under controlled ambient illumination (60 lux). Before testing, rats were allowed to acclimate to the experimental room for at least 30 min. Behavioral tests were performed in the sequence described below. The apparatus were cleaned with 10% ethanol between rats to minimize olfactory cues. Behavioral tests were conducted by two trained experimenters and video-recorded using a camera (D-link, model IP2P Wireless, Shenzhen, China) positioned above each apparatus. The recordings were subsequently analyzed by researchers blinded to group assignment.

#### 2.2.1 Anxiety-like behavior

Anxiety-like behavior was evaluated on the 115th and 116th days of the experiment using the open-field test and elevated plus-maze tests, respectively, as previously described by Soares et al. (2025) (33). In the open-field test, each rat was placed in the center of the arena and observed for 5 min. Locomotor activity, defined as the number of segments crossed with all four paws, as well as freezing, rearing, and grooming time, was recorded (37). In the elevated plus-maze, each rat was positioned on the central platform facing one of the open arms and observed for 5 min. The number of entries into the open and closed arms and the time spent in each arm were recorded (38).

#### 2.2.2 Depressive-like behavior

Depressive-like behaviors were assessed on the 117th day of the experiment using the splash test followed by the forced swim test, with a 1-h interval between tests. In the splash test, a 10% sucrose solution was sprayed onto the dorsal surface of each rat, and the total grooming time for 5 min (39). Subsequently, each rat was individually placed in a cylindrical tank (30 cm diameter × 50 cm height) filled with water to a depth of 30 cm and maintained at 25 ± 2 °C. Rats were subjected to a single 5 min session, during which the time spent swimming, climbing, immobile, and diving, as well as the number of dives, were recorded (40). The water was changed after each rat left.

### 2.3 . Insulin tolerance test

On the 118th day of the experiment, 48 h before euthanasia, rats underwent an insulin tolerance test (ITT) under basal conditions. Rats received an intraperitoneal injection of insulin (0.75 IU/kg body weight) diluted in sterile 0.9% saline solution, and blood glucose measurements were obtained at 0, 15, 30, 60, 90, and 120 min using a glucometer (Accu-Chek Performa, Jaguaré, Brazil) (41). The rate constant for plasma glucose disappearance (KITT) was calculated using the following equation:


$$\begin{array}{l} K_{ITT}=\left(\frac{0.693}{T_{1/2}}\right)\times 100 \end{array}$$

where *T*_1/2_ represents the time required for blood glucose levels to decrease by 50% from baseline values (42).

### 2.4 Euthanasia and biological sample collection

At the end of the 17-week experimental period, rats were fasted for 8 h and subsequently euthanized by guillotine decapitation (EB271, Insights, Ribeirão Preto, Brazil). Adipose tissue and brain were immediately collected for further analyses. Blood was collected into sterile tubes and centrifuged (1,040 × *g* for 10 min) to obtain serum for biochemical analyses.

### 2.5 Biochemical analyses

Serum insulin concentrations were measured using an enzyme-linked immunosorbent assay (ELISA) kit (BioVenic, EK11F267, Hauppauge, NY, USA), whereas serum leptin levels were quantified using an electrochemiluminescence immunoassay (Abcam, AB108881, Waltham, MA, USA) (32). Insulin resistance and pancreatic β-cell function were estimated using the Homeostatic Model Assessment of Insulin Resistance (HOMA-IR) and the Homeostatic Model Assessment of β-cell Function (HOMA-β), respectively (43).

### 2.6 Adiposity index and histological the brain and adipose tissue

Hippocampal and adipose tissue samples were processed using standard histological procedures, including dehydration in increasing alcohol concentrations, xylene clarification, paraffin embedding, and sectioning at 3 μm thickness. Hematoxylin and eosin (H&E) staining was performed according to standard protocols to evaluate tissue morphology. Images were captured using a microscope (Motif BA 200, Kowloon, Hong Kong) equipped with a 40 × objective lens. The histological assessment was performed by an investigator blinded to the experimental group allocation.

Hippocampal sections were evaluated across the major anatomical subregions, including CA1, CA2, CA3, CA4, and the dentate gyrus (DG), according to the classical organization of the hippocampal formation. The histological assessment focused on general tissue architecture and cellular morphology, including the organization and integrity of the hippocampal layers, cellular distribution and density, nuclear morphology, cytoplasmic characteristics, cellular rarefaction, vacuolization, and vascular alterations.

Adipocyte area was quantified in μm^2^ using ImageJ software (44). Up to 100 adipocytes per adipose tissue sample were analyzed. For morphometric analysis, representative microscopic fields were selected from each sample, avoiding overlapping adipocytes and tissue artifacts.

Mesenteric, retroperitoneal and epididymal adipose tissue were removed and weighed. The adiposity index was calculated and expressed as a percentage according to the following formula (45):


$$\begin{array}{l} Adiposity\;index\;\left(\%\right)=\;\frac{\begin{array}{c} (mesenteric\;+\\ \;retroperitoneal\;+\;epididymal\;fat) \end{array}}{Final\;body\;weigh}\times 100 \end{array}$$

Immunohistochemical analysis of IL-6 was performed on visceral adipose tissue sections using a peroxidase-based detection method. Endogenous peroxidase activity was blocked with 3% hydrogen peroxide for 5 min. The sections were subsequently incubated with a primary anti-IL-6 antibody (1:100 dilution; ab9324, Abcam, Cambridge, UK) for 60 min at room temperature. After washing, the samples were incubated with a horseradish peroxidase-conjugated polymer secondary antibody system for 10 min at room temperature. Immunoreactivity was visualized using 3,3′-diaminobenzidine tetrahydrochloride (DAB; Vector Laboratories, Burlingame, CA, USA) for 3 min, followed by hematoxylin counterstaining for 5 min. Images were acquired using an Eclipse 80i microscope (Nikon, Tokyo, Japan) under 400 × magnification. For morphometric analysis, twenty randomly selected, non-overlapping microscopic fields were captured from each sample using a digital camera coupled to the microscope. The area of positive IL-6 immunostaining was quantified using KS300 image analysis software (Zeiss, Zaventem, Belgium). Brown-stained pixels corresponding to positive immunoreactivity were identified and converted into binary images for quantitative analysis. Results were expressed as the immunopositive area (μm^2^), as previously described (46, 47). The IL-6-immunopositive area was used as a measure of tissue IL-6 immunoreactivity and was not interpreted as a direct measure of tissue IL-6 concentration.

The obesity parameters considered in this study were significant adipocyte hypertrophy, confirmed by histological and morphometric analyses, and the presence of metabolic and neurobehavioral alterations (48, 49).

### 2.7 Statistical Analysis

Data normality was assessed using the Shapiro–Wilk test. Normally distributed data were expressed as mean ± standard deviation. ITT data were analyzed using two-way analysis of variance (ANOVA), followed by Tukey's *post hoc* test. For variables evaluated at a single time point, comparisons between the control and obese groups (CG vs. OG) were performed using an independent-samples Student's *t*-test when parametric assumptions were met. Comparisons among the obese experimental groups (OG, OCR, and OTRF) were performed using one-way ANOVA followed by Tukey's *post hoc* test. For non-normally distributed data, the Mann–Whitney U test was used for CG vs. OG comparisons, whereas the Kruskal–Wallis test followed by Dunn's *post hoc* test was used for comparisons among OG, OCR, and OTRFA significance level of 5% (*p* < 0.05) was adopted for all analyses. Statistical analyses were performed using the free version of GraphPad Prism 11 (GraphPad Software, La Jolla, CA, USA).

## 3 Results

### 3.1 Caloric intake and weight changes

During the 13-week pre-intervention period, no differences were observed between the CG and OG groups in caloric intake (*p* > 0.05; Figure 2A) or body weight gain (*p* > 0.05; Figure 2B). During the intervention period, the OCR group exhibited a significantly lower caloric intake compared with the OG group (*p* < 0.05). No significant differences in caloric intake were observed between the OTRF and OG groups (*p* > 0.05) or between the OTRF and OCR groups (*p* > 0.05) (Figure 2C). Only the OCR group showed a significant reduction in body weight compared with the OG group during the intervention period (*p* < 0.05; Figure 2D). Despite the absence of a significant reduction in body weight in the OTRF group, both OCR and OTRF groups showed significantly lower adiposity index compared with the OG group (*p* < 0.05; Figure 2E). The reduction in adiposity index was more pronounced in the OCR group than in the OTRF group (*p* < 0.05).

**Figure 2:**
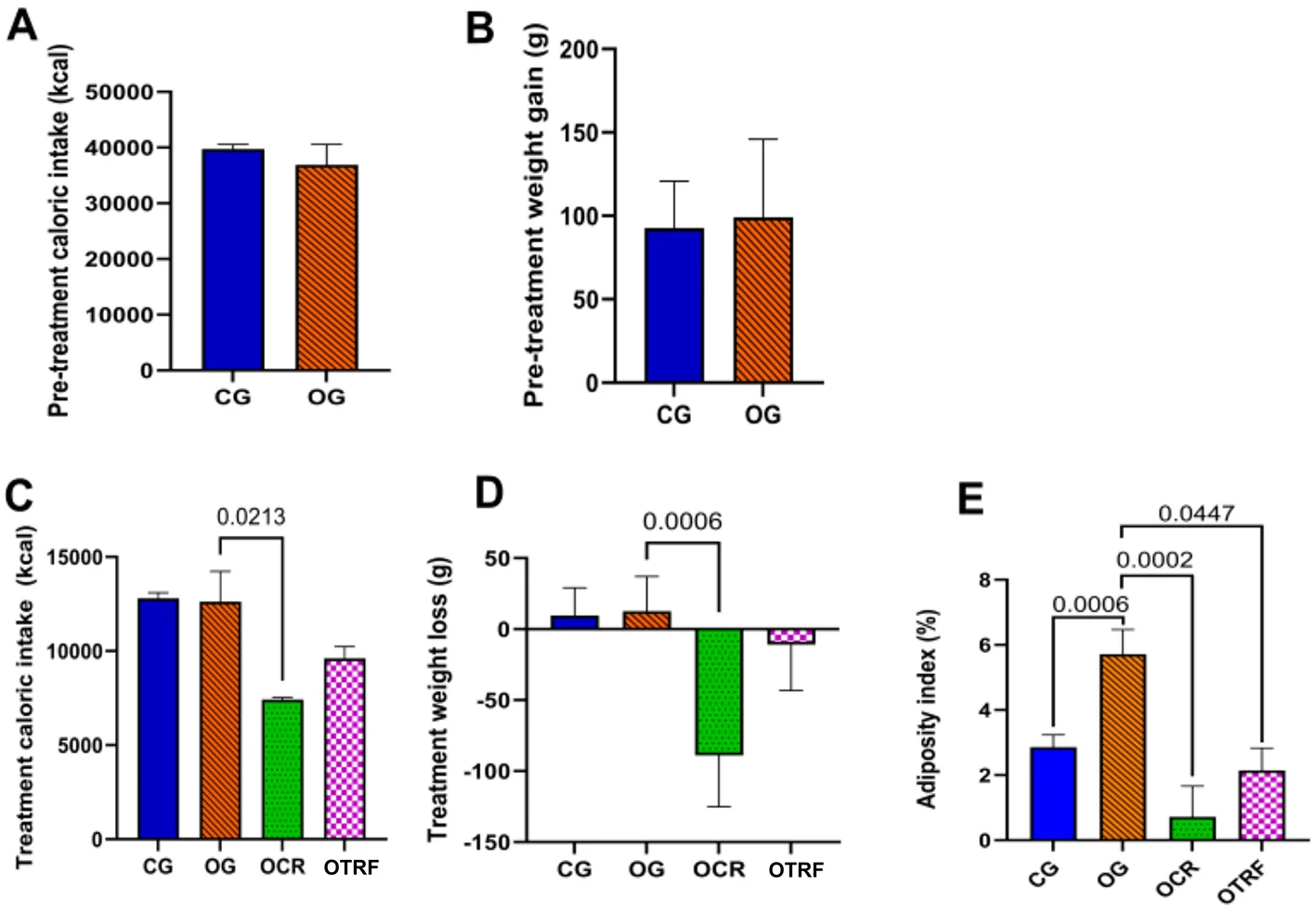
Caloric intake **(A)** and weight gain **(B)** during pre-intervention period; and caloric intake **(C)** and weight loss **(D)** during intervention period and adiposity index **(E)**. OC and OG groups are compared using: Student's *t*-test, *p* < 0.05. OG, OCR and OTRF groups are compared using: Treatment caloric intake: one-way ANOVA, *p* < 0.05, Tukey's hoc test; Treatment weight loss and adiposity index: Kruskal-Wallis test *p* < 0.05, Dunn's hoc test. CG, control group; OG, obese group; OCR, obese group submitted to caloric restriction; OTRF, obese group submitted to time-restricted feeding.

### 3.2 Anxiety-like behavior

Locomotor activity was reduced in rats fed the high-sugar diet (OG) compared to the CG group, whereas the other parameters evaluated in the open field and elevated plus-maze tests remained similar across groups (*p* > 0.05; Figures 3A–H). In contrast, both the OCR and OTRF groups exhibited increased locomotor activity and time spent in an upright posture (rearing), as well as reduced freezing time in the open field test, compared to rats in the OG group (*p* ≤ 0.05; Figures 3A–C). No significant differences in grooming time were detected among the OG, OCR, and OTRF groups (*p* > 0.05; Figure 3D).

**Figure 3:**
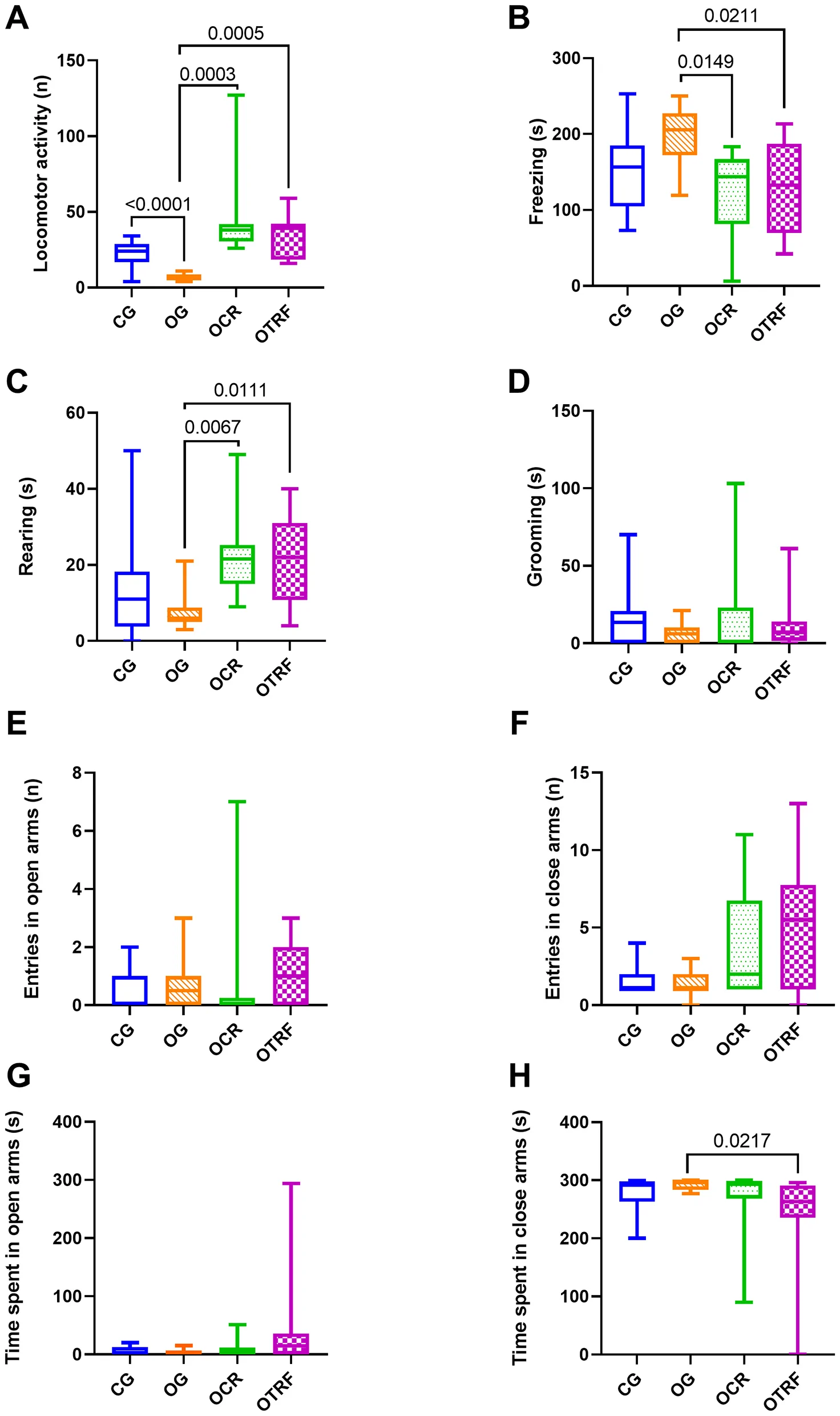
Behavioral parameters evaluated in the open field test **(A–D)** and the elevated plus maze **(E–H)** of experimental groups. CG and OG groups are compared using: Locomotor activity and freezing Student's *t*-test, *p* < 0.05; others: Mann-Whitney U test, *p* < 0.05. OG, OCR and OTRF groups are compared using: Freezing (s): one-way ANOVA, *p* < 0.05, Tukey's hoc test; Others: Kruskal-Wallis test *p* < 0.05, Dunn's hoc test. CG, control group; n: number; OG, obese group; OCR, obese group submitted to caloric restriction; OTRF, obese group submitted to time-restricted feeding; s, seconds.

In the elevated plus maze test, only the OTRF group exhibited reduced time spent in the closed arms compared with the OG group (*p* < 0.05; Figure 3H). No significant differences were observed between the OCR and OTRF groups in the parameters associated with anxiety-like behavior. Overall, these results indicate that 17 weeks of HSD consumption were not associated with increased anxiety-like behavior, whereas CR and TRF were associated with increased exploratory behavior in HSD-fed rats.

### 3.3 Depression-like behavior

Obese rats fed the HSD (OG group) exhibited shorter swimming time (*p* < 0.05; Figure 4A), shorter climbing time (*p* < 0.05; Figure 4B), and longer immobility time in the forced swim test (*p* < 0.05; Figure 4C), as well as a shorter grooming duration in the splash test, compared with control rats (*p* ≤ 0.05; Figure 4F). In contrast, OTRF rats displayed longer swimming time and a greater number of dives (*p* < 0.05; Figures 4A, E), together with reduced immobility time than OG rats (*p* < 0.05; Figure 4C). In addition, OTRF rats displayed greater active coping behavior than OCR-treated animals as indicated by longer swimming duration and a greater number of dives (*p* ≤ 0.05; Figures 4A, E). Diving time in the forced swim test did not differ significantly between groups (*p* < 0.05; Figure 4D).

**Figure 4:**
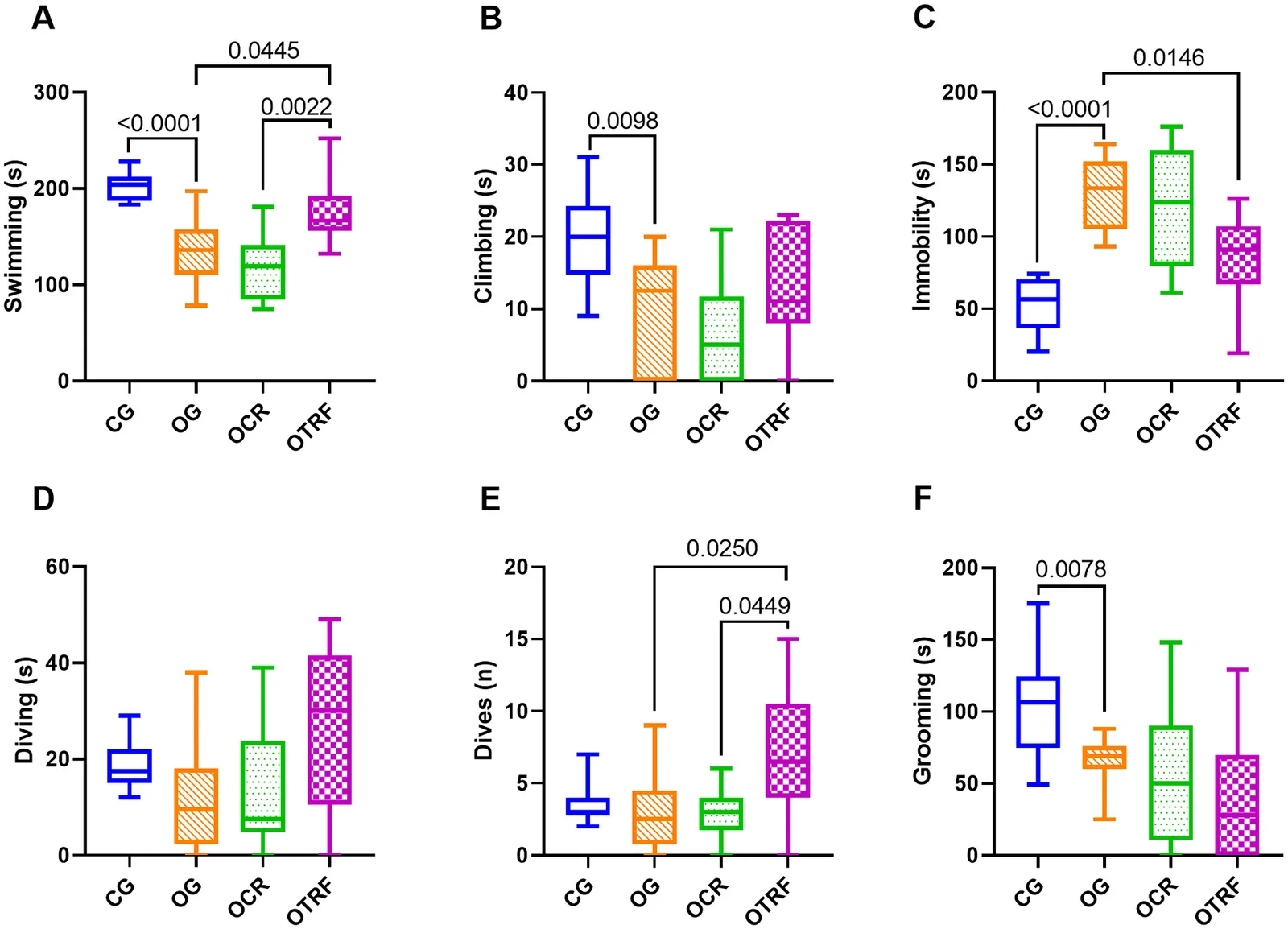
Depressive-like behavioral in forced swim **(A–E)** and splash **(F)** tests experimental groups. CG and OG groups are compared using: Dives: Mann-Whitney U test, *p* < 0.05; others: Student's *t*-test, *p* < 0.05. OG, OCR and OTRF groups are compared using: Dives(n): Kruskal-Wallis test *p* < 0.05, Dunn's hoc test; Others: one-way ANOVA, *p* < 0.05, Tukey's hoc test. CG, control group; *n*, number; OG, obese group; OCR, obese group submitted to caloric restriction; OTRF, obese group submitted to time-restricted feeding; s, seconds.

No significant differences were observed among the OG, OCR, and OTRF groups grooming time during the splash test (*p* > 0.05; Figure 4F). HSD consumption was associated with behavioral alterations consistent with a depressive-like behavioral phenotype, whereas OTRF was associated with greater active coping behavior in the forced swim test.

### 3.4 Effects of caloric restriction and TRF on glucose homeostasis, insulin sensitivity and leptin levels

In the insulin tolerance test (ITT), HSD-fed rats (OG group) exhibited higher blood glucose levels only 30 min after insulin administration compared with the CG group (*p* < 0.05; Figure 5A). Furthermore, the OG displayed impaired insulin sensitivity, as evidenced by an increased area under the curve (AUC) during the ITT (*p* < 0.05; Figure 5B) and a lower glucose disappearance rate (KITT) (*p* < 0.05; Figure 5C). Notably, only the CR intervention (OCR group) significantly reduced blood glucose levels throughout the ITT compared with both the OG and OTRF groups (*p* < 0.05; Figures 5A, B) and improved insulin sensitivity compared with the OG group, as demonstrated by a reduced AUC. Although no significant difference in KITT was observed between the OCR and OG groups (*p* > 0.05; Figure 5C).

**Figure 5:**
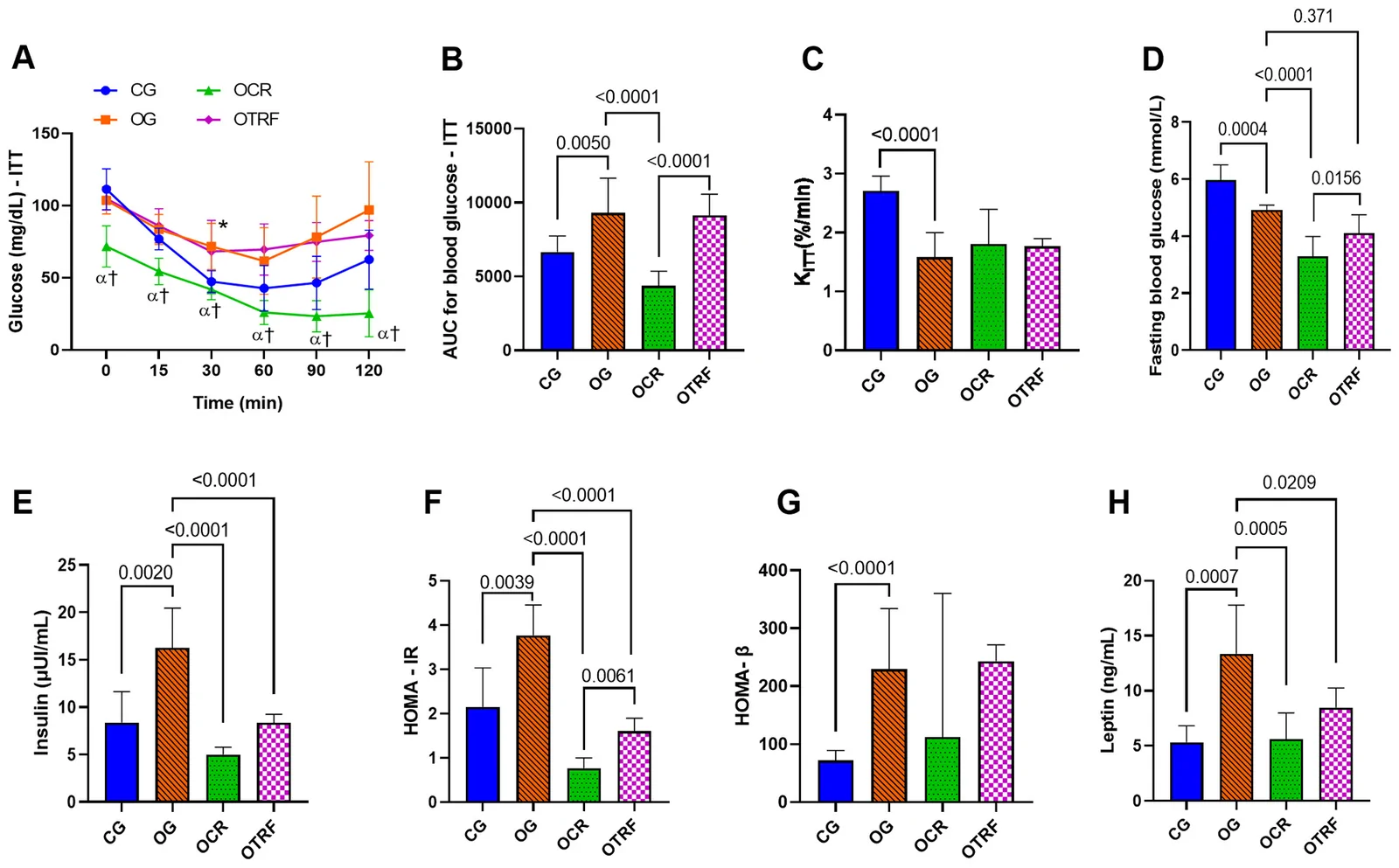
ITT **(A)**, AUC for blood glucose -ITT **(B)**, KITT **(C)**, fasting blood glucose **(D)**, insulin **(E)**, HOMA-IR **(F)**, HOMA-β **(G)**and leptin **(H)** of experimental groups. ITT: two-way ANOVA, *p* < 0.05, Tukey's hoc test. CG and OG groups are compared using: KITT : Mann-Whitney U test, *p* < 0.05; others: Student's *t*-test, *p* < 0.05.OG, OCR and OTRF groups are compared using: AUC ITT, fasting blood glucose, insulin, HOMA-IR, HOMA-β and leptin: one-way ANOVA, *p* < 0.05, Tukey's hoc test; KITT: Kruskal-Wallis test *p* < 0.05, Dunn's hoc test. CG, control group; OG, obese group; OCR, obese group submitted to caloric restriction; OTRF, obese group submitted to time-restricted feeding; AUC, area under the curve; ITT, insulin tolerance test; KITT, ITT glucose decay percentage constant; HOMA-IR, homeostasis model for insulin resistance; HOMA-β, homeostasis model for β cell function.

Interestingly, fasting glucose levels were lower in HSD-fed obese rats than in control animals (*p* < 0.05; Figure 5D), whereas serum insulin concentrations and HOMA-IR, and HOMA-β values were significantly higher in the OG group than in the CG group (*p* < 0.05 for all comparisons; Figures 5E, F), indicating alterations in glucose metabolism associated with HSD consumption. In contrast, reductions in fasting glucose, serum insulin, and HOMA-IR values were observed in the OCR and OTRF groups compared with the OG (*p* < 0.05 for all comparisons; Figures 5D–F). No significant differences in HOMA-β were observed between the OCR and OTRF groups (*p* > 0.05; Figure 5G). Furthermore, the OCR group exhibited more pronounced improvements in glucose metabolism than the OTRF group, as evidenced by greater reductions in AUC, fasting blood glucose, and HOMA-IR (*p* < 0.05; Figures 5B, D, and F).

The OG group also exhibited higher serum leptin concentrations than the CG group, whereas both the OCR and OTRF groups significantly reduced circulating leptin levels compared with the OG group (*p* < 0.05; Figure 5H).

Overall, caloric restriction (CR) and time-restricted feeding (TRF) attenuated the alterations in glucose metabolism and circulating leptin levels associated with HSD consumption. However, CR appeared to be more effective than TRF in improving glycemic parameters and insulin sensitivity in HSD-fed obese rats.

### 3.5 Effects of caloric restriction and TRF on hippocampal morphology, adipose tissue histomorphometry, and IL-6 immunoreactivity

No overt histopathological alterations were observed in the hippocampal sections of control rats (CG) or HSD-fed obese rats (OG) based on the qualitative assessment of H&E-stained sections (Figures 6A, B). Localized morphological alterations were observed in the OCR and OTRF groups (Figures 6C, D; arrows indicate the respective findings). However, given the qualitative nature of the assessment and the limitations of H&E staining, these observations were not interpreted as evidence of neuronal degeneration, neuronal injury, or neuroinflammation.

**Figure 6:**
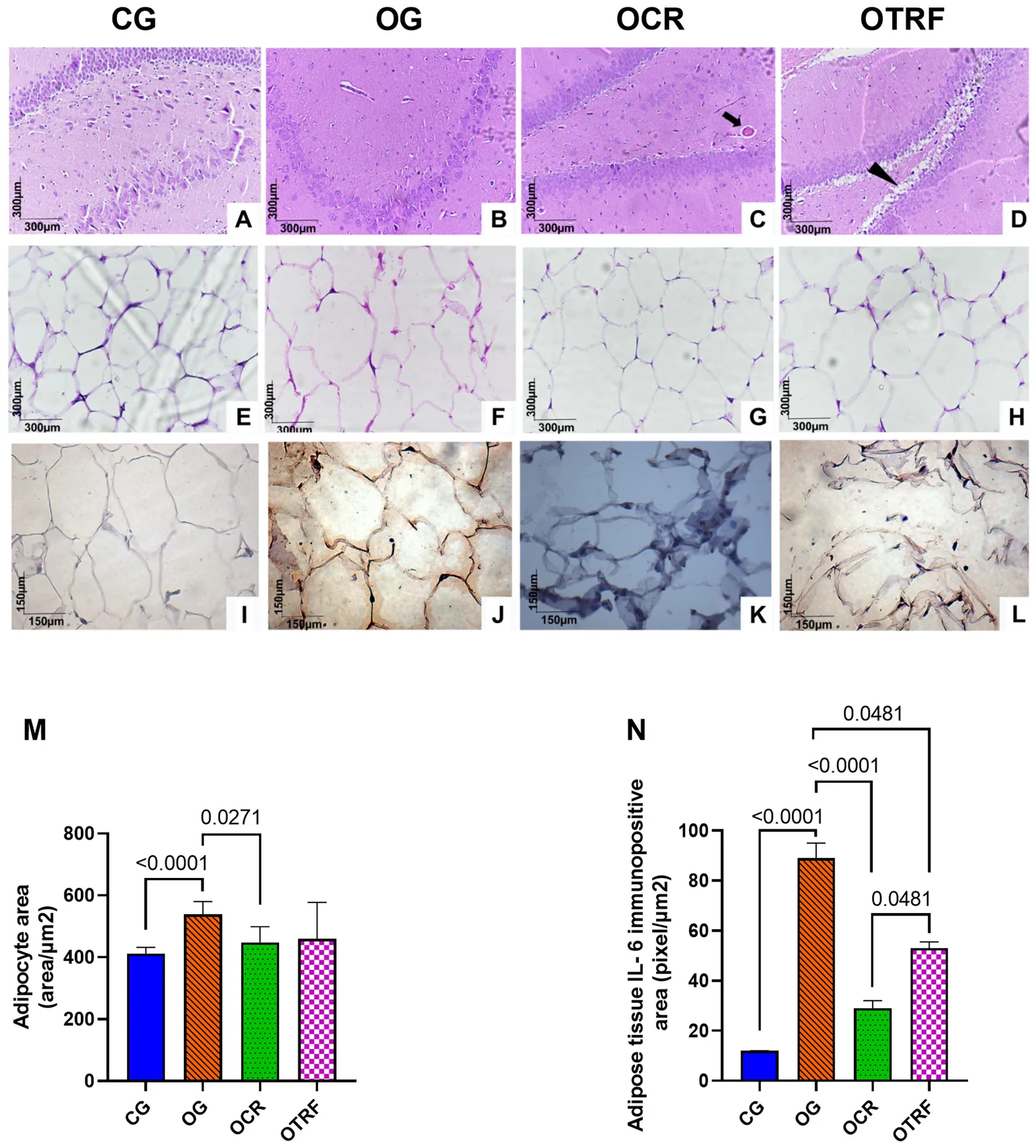
Hippocampus histology **(A-D)**, visceral adipose tissue histology **(E-H)**, representative images of IL-6 immunohistochemical staining **(I–L)**, adipocyte area **(M)** and adipose tissue IL-6-immunopositive area **(N)** of experimental groups. CG and OG groups are compared using: adipocytes size: Student's *t*-test, *p* < 0.05, others: Mann-Whitney U test, *p* < 0.05; OG, OCR and OTRF groups are compared using: adipocytes size: one-way ANOVA, *p* < 0.05, Tukey's hoc test; IL-6-immunopositive area: Kruskal-Wallis test *p* < 0.05, Dunn's hoc test. CG, control group; OG, obese group; OCR, obese group submitted to caloric restriction; OTRF, obese group submitted to time-restricted feeding.

The CG group exhibited adipocytes with relatively homogeneous size and morphology (Figure 6E). However, adipose tissue hypertrophy and a greater adipocyte area were observed in the OG compared with the CG (*p* < 0.05; Figures 6F, M), supporting the obese phenotype induced by HSD consumption. Among the interventions, only the OCR group exhibited a significant reduction in adipocyte area compared to the OG group (*p* < 0.05; Figures 6G–I, M).

Representative images of IL-6 immunostaining in adipose tissue are shown in Figures 6I–L. The OG group exhibited greater IL-6 immunostaining in adipose tissue than the CG group (Figure 6J) with a significantly larger IL-6-immunopositive area (*p* < 0.05; Figure 6N), indicating increased IL-6 immunoreactivity in adipose tissue. Both OCR and OTRF reduced IL-6 immunoreactivity (Figures 6K, L) and significantly decreased the IL-6-immunopositive area compared with the OG, with a more pronounced reduction observed in the OCR group than in the OTRF group (*p* < 0.05; Figure 6N).

Overall, HSD consumption was associated with adipose tissue hypertrophy and increased IL-6 immunoreactivity, whereas OCR and OTRF were associated with lower IL-6 immunoreactivity compared with OG, with a more pronounced reduction observed in OCR than in OTRF under the experimental conditions of this study.

## 4 Discussion

The present study compared the effects of CR and TRF on metabolic, behavioral, and tissue outcomes in HSD-induced obesity. HSD consumption resulted in metabolic and behavioral disturbances, whereas the responses to CR and TRF varied among the parameters evaluated. These differences indicate that reducing caloric intake and restricting the daily feeding window may have differential effects on the consequences of HSD consumption.

Despite prolonged HSD consumption, OG rats exhibited caloric intake and body weight gain comparable to those of CG rats. Body weight alone, however, may not adequately reflect changes in adiposity and body composition or the metabolic consequences of HSD consumption (50–52). Previous findings from our research group showed increased fat mass and reduced lean mass in OG rats (8), indicating changes in body composition despite similar body weight. Excessive consumption of carbohydrate-rich diets may also favor *de novo* lipogenesis and fat accumulation (50, 53), which could contribute to metabolic alterations even in the absence of greater body weight gain.

A previous study from our research group showed that OG rats exhibited increased fat mass and reduced lean mass (8). Excessive consumption of carbohydrate-rich diets may favor *de novo* lipogenesis and fat accumulation (50, 53). Increased adiposity, even in the absence of excess body weight, has been associated with adverse metabolic outcomes (52, 54, 55). Therefore, the similar body weight gain observed in CG and OG rats does not exclude possible differences in body composition. This interpretation is supported by the adipocyte hypertrophy observed in OG rats.

Among the intervention groups, only OCR rats showed a significant reduction in body weight compared with OG rats. However, the adiposity index was reduced in both OCR and OTRF rats, with a greater reduction observed in OCR rats. The more pronounced effect of CR on body weight may be related to the sustained reduction in energy intake, as OCR rats received 30% fewer calories than OG rats. In contrast, OTRF rats had *ad libitum* access to food during the feeding window, which may have allowed compensatory caloric intake after the fasting periods and contributed to the absence of a significant reduction in body weight.

After 17 weeks of HSD exposure, OG rats did not exhibit increased anxiety-like behavior compared with CG rats, contrasting with previous reports using high-sugar diets (56, 57). Differences in sugar composition, concentration, and duration of dietary exposure may contribute to these divergent findings (58). Although the HSD used in the present study was rich in mono- and disaccharides, its total carbohydrate content (72.71%) was slightly lower than that of the standard commercial diet (75.57%). Thus, the diets differed primarily in carbohydrate composition rather than total carbohydrate content, which may have contributed to the behavioral response observed.

Although OG rats did not exhibit increased anxiety-like behavior, they showed behavioral alterations consistent with a depressive-like phenotype, including reduced grooming in the splash test and reduced swimming and climbing, together with increased immobility, in the forced swim test. These behavioral changes occurred alongside hyperinsulinemia, increased HOMA-IR, and elevated circulating leptin levels in OG rats. Insulin resistance and alterations in adipokine signaling have been associated with mood-related behavioral changes in previous studies (59, 60), suggesting a possible link between the metabolic and depressive-like alterations observed here. However, the present study was not designed to establish a causal relationship between these outcomes.

Regarding the dietary interventions, OCR and OTRF rats showed increased ambulation and rearing and reduced freezing in the open-field test compared with OG rats, indicating greater locomotor and exploratory activity in a novel environment. Similar changes following CR and TRF have been reported in previous studies (33, 61–64). However, the open-field parameters evaluated are not specific measures of anxiety-like behavior, and the primary elevated plus-maze outcomes did not consistently support an anxiolytic-like effect of either intervention. Therefore, the open-field test findings are better interpreted as changes in locomotor and exploratory behavior rather than evidence of an anxiolytic-like effect. The reduced time spent in the closed arms by OTRF rats in the elevated plus-maze test should likewise be interpreted cautiously as potentially reflecting increased exploratory activity rather than an anxiolytic-like effect.

The effects of OTRF were more evident in the assessment of depressive-like behavior. Compared with OG rats, OTRF rats showed increased swimming, reduced immobility, and a greater number of dives in the forced swim test. Similar improvements following TRF have been reported in diabetic, healthy, and shift-work stress rodent models (33, 65, 66). Changes in neurotransmitter signaling and inflammatory pathways have been proposed as possible mechanisms in these models (33, 65, 66), but these pathways were not evaluated in the present study. The sequence of behavioral testing should also be considered when interpreting the behavioral findings, as previous behavioral experience may influence performance in subsequent tests (67). Although the open-field test and elevated plus-maze were performed on separate experimental days, with approximately 24 h between assessments, the possibility that prior exposure to the open-field test influenced subsequent elevated plus-maze performance cannot be completely excluded.

HSD consumption was associated with metabolic alterations in OG rats, including hyperinsulinemia, increased HOMA-IR, and elevated circulating leptin levels, consistent with impaired insulin homeostasis. High-sugar diets, particularly those rich in fructose and sucrose, have been associated with impaired insulin action and altered glucose homeostasis in rodent models (68, 69). Elevated circulating leptin is also commonly associated with increased adiposity and metabolic dysfunction in obesity (70). A recent systematic review and meta-analysis showed that metabolic alterations induced by high-sugar feeding in rodents may occur without a corresponding increase in body weight (71), consistent with the metabolic phenotype observed in OG rats.

OCR rats showed improvements in several metabolic alterations observed in OG rats, with lower ITT AUC, fasting insulin, HOMA-IR, and circulating leptin levels. OTRF rats also exhibited lower fasting glucose, insulin, HOMA-IR, and leptin levels compared with OG rats; however, these changes were not accompanied by a significant reduction in ITT AUC. This suggests that the improvement in fasting metabolic parameters observed in OTRF rats was not accompanied by a comparable change in the response to exogenous insulin. Beneficial effects of CR and TRE/TRF on glucose homeostasis, insulin-related parameters, and circulating leptin have also been reported in human and rodent studies (34, 72–74). Differences in the magnitude of these responses may depend on intervention duration, fasting and feeding schedules, and the timing of food availability relative to the active and inactive phases (60, 61).

No overt hippocampal morphological alterations were observed in OG rats, whereas localized changes were identified in both intervention groups. Similar findings have been reported in rats subjected to fasting interventions in the absence of diet-induced obesity. In that study, TRF was associated with pyknotic neurons and increased gemistocytic astrocytes in the hippocampus, together with greater hippocampal NF-κB immunoreactivity compared with control rats (33). The occurrence of these changes following TRF in non-obese rats suggests that they may not be exclusively related to obesity. In the same study, fasting interventions were also associated with improvements in some depressive-like behavioral parameters despite the presence of hippocampal morphological alterations.

In the present study, OTRF rats showed improvements in depressive-like behavioral parameters despite the presence of localized hippocampal morphological alterations. The relationship between these behavioral and histological findings remains unclear. H&E staining allowed the identification of morphological changes but, in the absence of complementary neuronal, glial, or inflammatory markers, their nature and functional significance cannot be determined.

Adipocyte hypertrophy was observed in OG rats, consistent with adipose tissue remodeling commonly observed in obesity (75). Among the intervention groups, OCR rats showed a significant reduction in adipocyte size, whereas no significant reduction was observed in OTRF rats. Adipocyte size is closely related to metabolic function, with larger adipocytes being associated with impaired insulin sensitivity and higher circulating leptin levels, independently of body weight (76, 77). In OCR rats, the smaller adipocytes occurred alongside a lower adiposity index and improvements in insulin-related parameters and circulating leptin. The concurrence of these morphological and metabolic changes suggests that adipose tissue morphology provides information on the response to CR that is not fully reflected by changes in body weight alone.

OG rats also exhibited increased IL-6 immunoreactivity in adipose tissue, whereas lower immunoreactivity was observed in both OCR and OTRF rats. Adipose tissue is an important source of IL-6, and increased IL-6 levels have been associated with adiposity and metabolic dysfunction in obesity. However, the relationship between IL-6 and insulin resistance is complex and varies according to the tissue and metabolic context (78, 79). In OCR rats, lower IL-6 immunoreactivity accompanied the reduction in adipocyte size, whereas OTRF rats exhibited lower IL-6 immunoreactivity without a significant change in adipocyte size. Therefore, changes in IL-6 immunoreactivity and adipocyte morphology did not occur in parallel in the two intervention groups. Because IL-6 was the only inflammatory marker assessed in adipose tissue, these findings should not be interpreted as evidence of a broader change in adipose tissue inflammation.

The absence of overt hippocampal alterations in the OG group indicates that HSD consumption did not produce detectable histopathological changes under the conditions assessed. The localized morphological findings observed in the intervention groups should be interpreted cautiously, as they were identified by H&E staining and were not supported by specific markers of neuronal or glial injury. The lack of specific neuronal and glial markers limits the extent to which subtle neuroinflammatory, synaptic, or neuronal alterations can be excluded.

Several limitations should be acknowledged. The exclusive use of male Wistar rats limits the generalizability of the findings. In addition, physical activity was not directly monitored, and food was provided during the inactive phase in the TRF protocol, which may have influenced the metabolic response to the intervention. Behavioral tests were performed at a single time point; therefore, the long-term effects of CR and TRF on behavior remain unknown. In addition, the CR and TRF protocols required daily dietary manipulation, which may have resulted in greater or different levels of investigator–animal interaction compared with the control and obese groups. Although all animals were handled according to standardized procedures, this difference in experimental handling could have influenced behavioral responses and therefore represents a potential confounding factor that cannot be excluded. Brain histology was evaluated using H&E staining, without additional markers to further characterize the morphological alterations observed in the hippocampus. Other brain regions involved in the regulation of anxiety- and depression-related behaviors, such as the prefrontal cortex and amygdala, as well as markers related to neuronal and inflammatory processes, including brain-derived neurotrophic factor (BDNF), TNF-α, and other inflammatory cytokines, were not evaluated. These additional analyses could help clarify the relationship between the behavioral and histological findings. IL-6 was the only inflammatory marker evaluated in adipose tissue, which limits a broader interpretation of the inflammatory response in this tissue. Additional inflammatory markers and immune cell characterization would provide a more comprehensive assessment of the adipose tissue response to CR and TRF.

Despite these limitations, the direct comparison between CR and TRF allowed their effects to be compared within a common experimental framework. The integration of metabolic, behavioral, and tissue assessments also provided a broader evaluation of the responses to these dietary interventions. However, the limitations discussed above should be considered when interpreting the behavioral, histological, and inflammatory findings.

## 5 Conclusion

Taken together, our findings indicate that CR was particularly effective in improving the metabolic alterations associated with HSD-induced obesity, whereas TRF was associated with greater active coping behavior in the forced swim test, as indicated by increased swimming and reduced immobility. However, these behavioral changes in OTRF rats coexisted with localized morphological alterations in the hippocampus. The biological significance of these hippocampal alterations remains unclear and requires further investigation.

## Data Availability

The original contributions presented in the study are included in the article/Supplementary material, further inquiries can be directed to the corresponding author.
