# Supplementary material for: Caloric restriction and time-restricted feeding differentially modulate metabolic, adipose tissue, and neurobehavioral outcomes in obese rats fed a high-sugar diet
Source: Front Nutr. 2026 Oct 29;13:1933559. doi: 10.3389/fnut.2026.1933559 (PMC13755932; doi:10.3389/fnut.2026.1933559)
Supplement: Supplementary file 1 [file Table_1.docx]

Supplementary Material

**Supplementary Table S1.** Ingredients, sugars quantified and macronutrients of the control diet and high-sugar diet (HSD) which used in the experiment (8).

| **Ingredients and composition** | **Diets** | |
| --- | --- | --- |
|  | **Control** | **HSD** |
| **Ingredients** |  |  |
| Crushed feed (g) | 100.00 | 45.20 |
| Refined sugar (g) | - | 9.60 |
| Commercial condensed milk (mL) | - | 45.20 |
| **Mono and disaccharides (mg/g)** |  |  |
| Glucose | 1.89 ± 0.09 | 37.64 ± 0.93* |
| Fructose | 0.83 ± 0.15 | 28.82 ± 0.24* |
| Maltose | 0.13 ± 0.02 | 11.30 ± 1.64* |
| Total sugars (mg/g) | 2.85 | 77.76 |
| **Macronutrients** (%) |  |  |
| Carbohydrate | 75.57 ± 0.14 | 72.71 ± 0.16* |
| Fat | 2.43 ± 0.01 | 4.29 ± 0.01* |
| Protein | 22.00 ± 0.10 | 23.00 ± 0.10* |
| Energy value (kcal) | 412.15 | 421.45 |

* Significant difference between the diets (Student's t-test, p≤0.05); HSD= high-sugar diet

Reference

8. de Souza DM, Cavalcante HC, dos Santos Lima M, Alves AF, da Veiga Dutra ML, D’Oliveira AB, et al. Intermittent fasting associated with coconut oil (Cocos nucifera L.) alters gut-liver axis parameters in diet-induced obese rats. Nutrition. 2024 May 1;121. doi:10.1016/j.nut.2024.112370 PubMed PMID: 38401196.
